# Growth and Characterization of CuO Nanostructures on Si for the Fabrication of CuO/p-Si Schottky Diodes

**DOI:** 10.1155/2013/126982

**Published:** 2013-05-23

**Authors:** S. Çetinkaya, H. A. Çetinkara, F. Bayansal, S. Kahraman

**Affiliations:** Department of Physics, Faculty of Arts and Sciences, Mustafa Kemal University, 31034 Hatay, Turkey

## Abstract

CuO interlayers in the CuO/p-Si Schottky diodes were fabricated by using CBD and sol-gel methods. Deposited CuO layers were characterized by SEM and XRD techniques. From the SEM images, it was seen that the film grown by CBD method is denser than the film grown by sol-gel method. This result is compatible with XRD results which show that the crystallization in CBD method is higher than it is in sol-gel method. For the electrical investigations, current-voltage characteristics of the diodes have been studied at room temperature. Conventional *I*-*V* and Norde's methods were used in order to determine the ideality factor, barrier height, and series resistance values. It was seen that the morphological and structural analysis are compatible with the results of electrical investigations.

## 1. Introduction 

Metal oxide nanoparticles have attracted considerable attentions in the last decades. Among them cupric oxide- (CuO-) based materials have various technological applications in ceramics, sensors, catalysis, batteries, solar cells, magnetic storage media, semiconductors, capacitors, diodes, and so forth [1–9] because of their novel mechanical, electronic, magnetic, and optical properties compared with those of conventional bulk materials. CuO is an important low-cost and nontoxic transition metal oxide with a narrow bandgap of ~1.2 eV at room temperature. A number of different techniques such as heating copper sheets in O_2_ atmosphere, immersing CuO sheets into ammonia or sodium hydroxide solutions, electrodepositing Cu(II) ions and sol-gel deposition [10–13] have been used to control size and morphology of CuO nanomaterials. Among these techniques, chemical bath deposition method (CBD)—a wet-chemical method—and sol-gel deposition technique are promising techniques because they are both simple, safe, environmental friendly, suitable for mass production, low temperature compatible, and cost-effective solution-based growth techniques. 

There has been considerable interest in the experimental studies of metal-semiconductor (MS) and metal-interlayer-semiconductor (MIS) type Schottky diodes in the past decades [14, 15]. The performance and quality of the Schottky diodes are especially dependent on the production conditions and formation of interlayer which strongly influence the device parameters such as ideality factor, barrier height, and serial resistance. Some of these device parameters can be manipulated by modified interfacial layers that are grown in between metal and semiconductor. There are few studies on MIS structures where copper oxide is used as interfacial layer in the Schottky diodes [9, 16, 17]. More studies are needed to improve the device parameters.

In this work, we report simple routes for the production of Au/CuO/pSi/Al MIS Schottky structures in which the insulating layers were grown by CBD and sol-gel methods. This is the first report which compares the effect of CuO layers obtained by CBD and sol-gel methods. The morphology of CuO nanostructures were investigated by scanning electron microscopy (SEM). We have also investigated the crystal structure by X-ray diffraction spectroscopy and diode parameters by conventional current-voltage measurements. 

## 2. Experimental Details

### 2.1. Materials

All the chemical reagents used in the experiments were analytical grade and were used without further purification. Copper(II) chloride dehydrate (CuCl_2_·2H_2_O), sulfuric acid (H_2_SO_4_), hydrogen peroxide (H_2_O_2_), hydrofluoric acid (HF), hydrochloric acid (HCl), polyethylene glycol (PEG4000), and diethanolamine (DEA) were purchased from Sigma-Aldrich Co. Ammonia solution (NH_4_OH), acetone (CH_3_COCH_3_), methanol (CH_3_OH), and ethanol (C_2_H_6_O) were purchased from Merck KGaA. P-type Si wafers (boron doped, resistivity: 1–10 Ω-cm, thickness: 400 *μ*m) were purchased from Si-Mat. Au and Al metals were purchased from Kurt J. Lesker Co.

### 2.2. Preparation of MIS Structures

The samples were prepared on a mirror cleaned and polished p-type Si wafer with (100) orientation and 1–10 Ω-cm resistivity. First, the wafer was chemically cleaned using the RCA1 and RCA2 cleaning procedures. The ohmic contact was made by evaporating Al on the back of the wafer in vacuum system of 10^−5^ Torr, followed by a temperature treatment at 573°C for 3 min in N_2_ atmosphere. The native oxide on the front surface of the wafer was removed in HF : H_2_O (1 : 10) solution and finally the wafer was rinsed in deionized water for 30 s before forming CuO layer. Two methods are used for the synthesis of the films onto the polished side of the Si substrates: the first method is CBD method. Back sides of the samples (ohmic side) were covered with an organic layer (photoresist) in order to prevent the growth of CuO on top of this side. 1.705 g copper(II) chloride dehydrate (CuCl_2_·2H_2_O) was dissolved in 100 mL deionized water under stirring in a magnetic stirrer at room temperature to obtain 0.1 M copper chloride solution. The solution was stirred for 15 min to ensure that CuCl_2_ dissolved completely. Then, pH value of the solution was increased to ~10.0 by adding aqueous ammonia under constant stirring. A blue solution of Cu(OH)_2_ was soon produced. Previously cleaned substrates were immersed into the solution. Then the solution was started to boil at ~90°C in order to convert Cu(OH)_2_ into CuO. Heating rate was 10°C/min, and it took about 10 min to boil the solution. After 5 min boiling, the substrates were taken out from the bath and washed with deionized water. To remove the back side organic layer, the substrates were washed with acetone and water. Finally, the substrates they were dried in air at room temperature for a day. The second method is sol-gel method. 0.512 g copper(II) chloride dehydrate was dissolved in 10 mL ethanol to obtain 0.3 M copper chloride solution. Then 0.5 mL DEA and 0.005 gr PEG4000 were added to the solution under stirring in a magnetic stirrer at room temperature. Then the front sides (polished side) of the samples were covered with this solution in a spin-coating system. After coating the substrates were dried in a tube furnace at 120°C for 5 min. This cycle (spin-coating and drying) was repeated for 6 times. Finally the substrates were annealed at 500°C for 60 min in order to remove volatile chemicals from the surface. After these processes, both of the samples (CBD and sol-gel) were placed in a physical vapor deposition (PVD) system. Au was evaporated through a shadow mask in a vacuum of 10^−5^ Torr. In this way, Au/CuO/p-Si/Al metal-insulator-semiconductor (MIS) structures were obtained. The areas of circular Schottky contacts were adjusted to 7.85 × 10^−3^ cm^2^.

### 2.3. Characterization of the Samples

Crystal structure of the films was examined by a Rigaku Ultima-IV X-ray diffractometer (XRD) (Cu K*α* radiation, *λ* = 1.540056 Å). A scan rate 0.05°/min was applied to record the patterns in the 2*θ* range of 30–80°. A JEOL JSM-5500LV scanning electron microscope (SEM) was operated at an acceleration voltage of 10 and 20 kV for morphological imaging. A computer interfaced Keithley 6487 Picoammeter/Voltage Source was used to investigate the diode parameters of the samples.

## 3. Results and Discussion

### 3.1. Morphological and Structural Characterization of CuO Films

**Figure 1:**
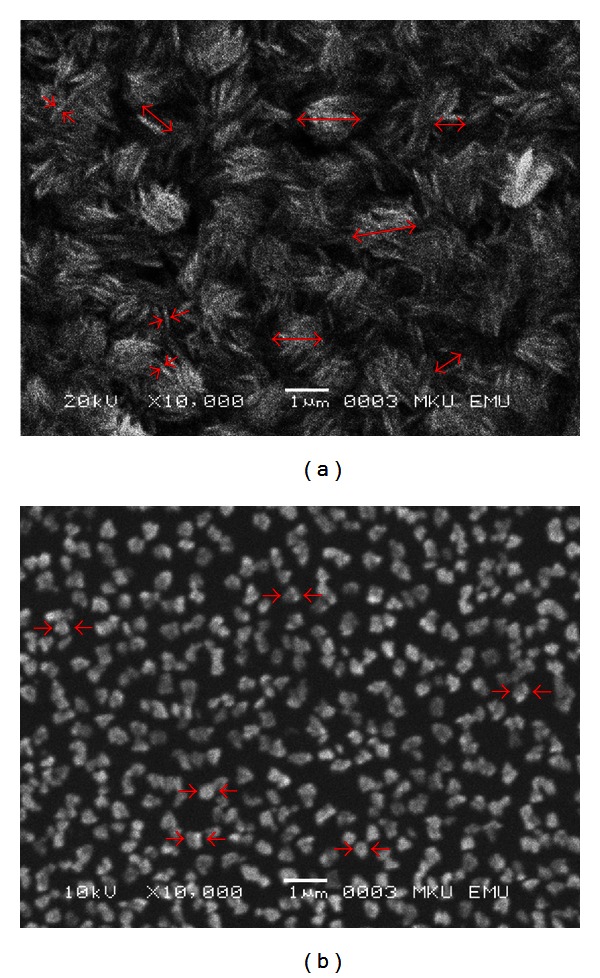
SEM images of the CuO films growth by the (a) CBD method and (b) sol-gel method.

Morphology of the films was investigated by scanning electron microscopy. As seen from Figure 1, CuO nanostructures were grown in two different shapes on p-Si substrate.  Figure 1(a) shows the SEM image of the nanostructures that were grown by CBD method. From this figure, it was seen that the needle-like nanostructures were firmly clasped together, thus formed clusters on the surface. Average thickness, length, and cluster diameter of the nanostructures were found to be ~97, 710, and 870 nm, respectively. On the other hand, Figure 1(b) shows the SEM image of the nanostructures that were grown by sol gel method. As seen from the figure the nanostructures were formed as nearly cubic shaped. Contrary to Figure 1(a), the structures were grown individually, that is, they are not connected to each other. This discreetness will affect the ideality factors and the barrier heights of the MIS structures that will be explained in the following section. The average diameter of the structures was found to be 340 nm. From these two figures, it is obviously seen that the growth method has a deep impact on the morphology of the structures. 

**Figure 2:**
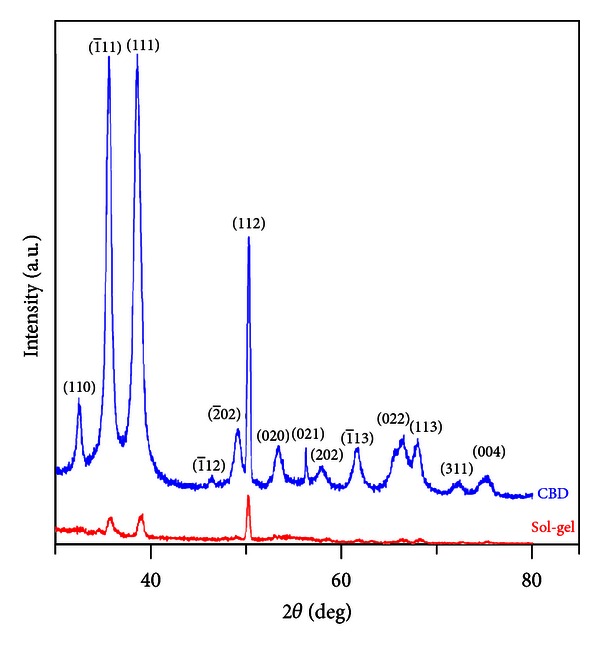
XRD patterns of the CuO films.

The crystal structures of the films were examined by an X-ray diffractometer. The XRD patterns of CuO films grown on the Si substrate are shown in Figure 2. All diffraction peaks can be clearly indexed to monoclinic CuO phase with lattice constants of *a* = 4.680 Å, *b* = 3.431 Å, *c* = 5.136 Å, and *β* = 99.26° (reference code: 01-080-0076). There were no peaks referred to Cu(OH)_2_ and/or CuCl_2_ which means the grown films completely consist of only CuO molecules or Cu(OH)_2_ and/or CuCl_2_ may be present in small extent and/or accumulated along grain boundaries of the crystallites constituting the film. XRD pattern also provides information on crystal orientations: the Miller-indexed ($\bar{1}$11), (111), and (112) reflections are the strongest, which indicate that they are preferential crystal planes of both of the films that were grown by CBD and sol-gel methods. From the XRD patterns, it can be deduced that the crystallization is stronger in CBD method than sol-gel method. From the SEM images it was seen that the needle-like nanostructures (Figure 1(a)) were covering the entire surface, but the cube-shaped nanostructures (Figure 1(b)) have some spaces among them. These SEM images also support the XRD results. Average grain size of the films were calculated by the Scherrer formula [18, 19]:

(1) $$\begin{array}{c} D=\frac{0.9\lambda }{\beta \cos\theta }, \end{array}$$

where *D* is the average grain size, *λ* is the X-ray wavelength of 0.1540056 nm, *β* is the full width at half maximum (FWHM) in radians, and *θ* is the diffraction angle. Each XRD peak obtained from a diffractometer may be broadened due to instrumental and physical factors (grain size, lattice strains, and dislocations). The microstrain (*ε*) and dislocation density (*ρ*) for the films were calculated by using the following formulas [18, 20]:

(2) $$\begin{array}{c} \varepsilon =\frac{\beta \cos\theta }{4},\\ \rho =\frac{15\varepsilon }{\alpha D}, \end{array}$$

where *α* is lattice constant. Changes in structural parameters were summarized in Table 1 for both of the films. It is seen from Table 1 that the grain sizes in both methods are close to each other, but still there is a small difference between them; this may be because of the reaction time. In CBD method, the reaction took about 20 min., but in sol-gel method, the reaction for every cycle took about 30 s. Big grain size caused big microstrain and big dislocation density values. This result means that the structure in sol-gel method has lower energy and thus, it is more stable. This may be the result of annealing processes in the sol-gel method.

### 3.2. Current-Voltage Characteristics of MIS Structures

When a metal/semiconductor contact with a thin interfacial layer is considered, it is assumed that the forward bias current of the device is due to thermionic emission current, and it may be expressed as [21]

(3) $$\begin{array}{c} I=I_{0}\exp\left(\frac{qV}{nkT}\right)\left[1-\exp\left(-\frac{qV}{kT}\right)\right], \end{array}$$

where *V* is the applied voltage, *k* is the Boltzmann constant, *T* is the temperature, and *I*_0_ is the reverse saturation current derived from the straight line intercept of ln⁡⁡*I* at *V* = 0 and is given by

(4) $$\begin{array}{c} I_{0}=AA^{*}T^{2}\exp\left(-\frac{q\Phi_{b,0}}{kT}\right), \end{array}$$

where Φ_*b*,0_ is the barrier height at zero bias (6), A* is the effective Richardson constant and equals to 32 A·cm^−2^·K^−2^ for p-type Si, *A* is the diode active area, and *n* is the ideality factor which is a measure of conformity of the diode to pure thermionic emission, and it is determined from the slope of the straight line region of the forward bias ln⁡⁡*I*-*V* characteristics. According to (1), *n* can be written as:

(5) $$\begin{array}{c} n=\frac{q}{kT}\frac{dV}{d\left(\ln I\right)}, \end{array}$$

(6) $$\begin{array}{c} q\Phi_{b,0}=kT\ln\left(\frac{AA^{*}T^{2}}{I_{0}}\right), \end{array}$$

**Figure 3:**
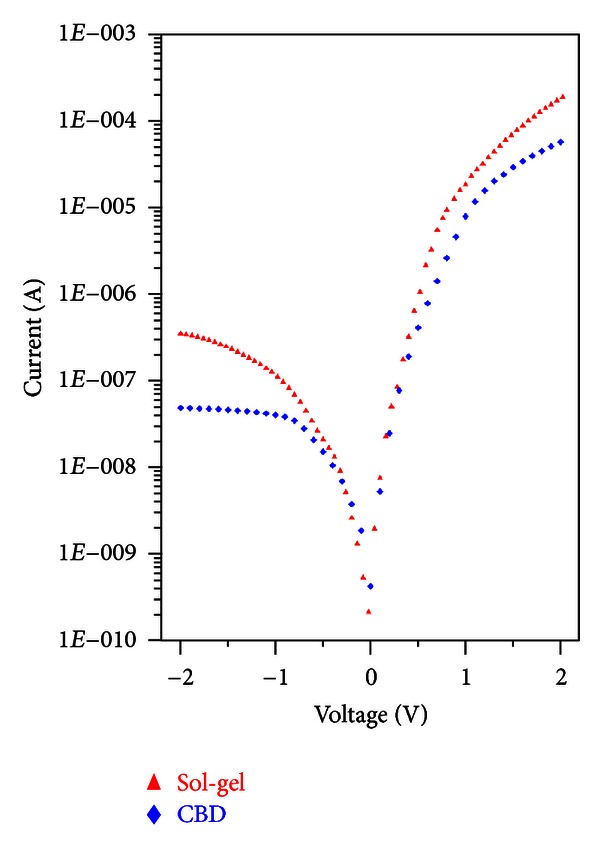
*I*-*V* characteristics of the Au/CuO/p-Si/Al MIS structures.

Figure 3 shows the semilogarithmic plot of forward-bias *I*-*V* characteristics of the Au/CuO/p-Si/Al structures at room temperature. The ideality factors of the MIS structures were calculated by (5) from the linear region of the forward-bias *I*-*V* plots. They were found as 2.80 and 1.58 for the structures grown by CBD and sol-gel methods, respectively. As understood from these values the diode deviates from the ideality because of the interfacial layer. From the SEM images and XRD patterns, it was seen that the CuO film grown by CBD method was homogenously covering the entire sample and the crystallinity of the film was very high. Both of these reasons caused the diode to deviate from the unity. On the other hand, the film grown by sol-gel method weakly covered the surface, and there are big empty regions among them. These are thought as the main reasons for the small ideality factor with respect to the other sample. In both cases the ideality factors are deviated from the unity which can be related to current mechanism of the structure, barrier height inhomogeneity, recombination-generation, series resistance and image force levering which is voltage dependent, and/or an interface oxide layer [22]. The values of the barrier heights of the structures were found to be ~0.79 eV for both of the structures from the *y*-axis intercepts of the semilog-forward-bias *I*-*V* plots and (6). 

**Figure 4:**
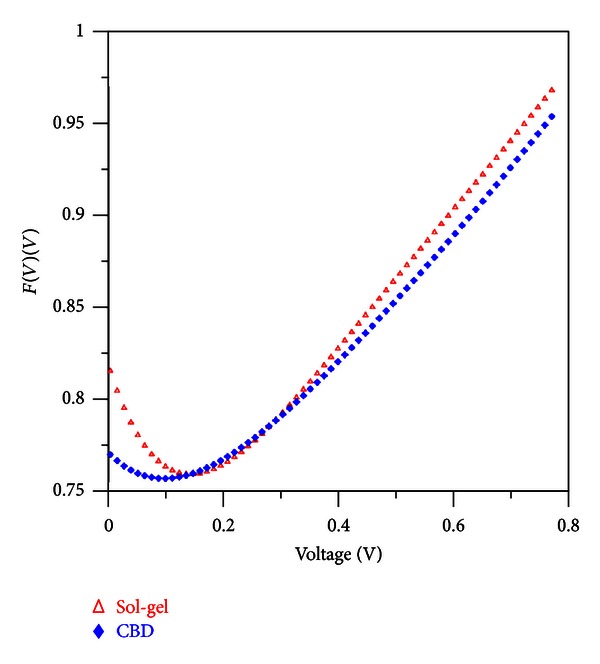
*F*(*V*) versus *V* plots obtained from the experimental *I*-*V* data in Figure 3.

In order to find the series resistance values, we have used the Norde's functions [22]. The function is defined as

(7) $$\begin{array}{c} F\left(V\right)=\frac{V}{\Upsilon }-\frac{kT}{q}\ln\left(\frac{I\left(V\right)}{AA^{*}T}\right), \end{array}$$

where *Υ* is the first integer greater than *n* and *I*(*V*) is the current obtained from the *I*-*V* curve. Once the *F*(*V*) versus *V* graph is obtained, the barrier height can be found by

(8) $$\begin{array}{c} \Phi_{b}=F\left(V_{0}\right)+\frac{V_{0}}{\Upsilon }-\frac{kT}{q}, \end{array}$$

where *F*(*V*_0_) is the minimum point of *F*(*V*) and *V*_0_ is the corresponding voltage. The Norde plots for the diodes are shown in Figure 4. The values of the series resistances have been calculated from Norde's function for each of the diodes by using the following relation:

(9) $$\begin{array}{c} R_{s}=\frac{kT\left(\Upsilon -n\right)}{qI}. \end{array}$$

By using Norde's method the series resistance values are found to be ~119 and 410 k*Ω* and the barrier heights are found to be 0.76 and 0.80 eV, for the structures obtained by CBD and sol-gel methods, respectively. Both the series resistance and barrier height values of the structure grown by CBD method are lower than the structure grown by sol-gel method. From these values, it is seen that the barrier height values calculated from *I*-*V* and Norde's methods are close to each other in the case of sol-gel method, but in CBD method, these values are not close to each other. In *I*-*V* method, the data only in the linear region (forward bias) is used for the calculations, but in Norde's method all the data in the forward region is used. From Figure 4, it can be obviously seen that the linear region in sol-gel process is clearer than the region in CBD method. This linearity causes the barrier height values found by *I*-*V* and Norde's methods to be close to each other. The ideality factors, barrier heights, and serial resistance values are listed in Table 2.

**Figure 5:**
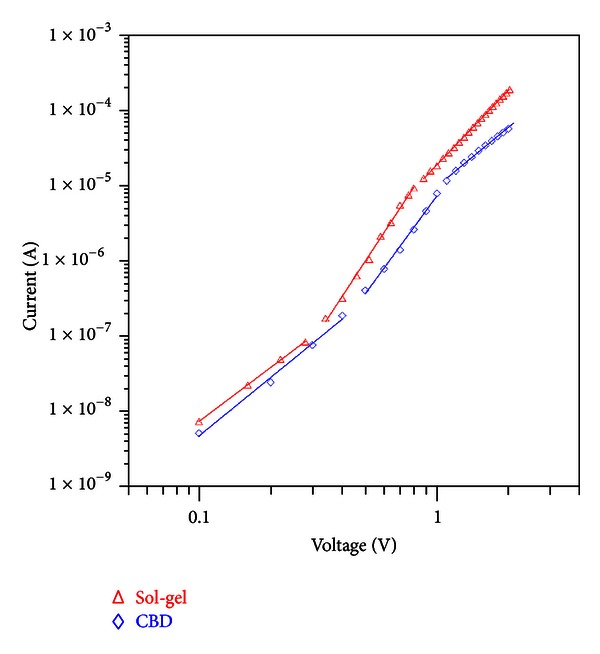
The forward bias log⁡(*I*) versus log⁡(*V*) plots of the Au/CuO/p-Si/Al diodes from the data in Figure 3.

Figure 5 shows the forward bias logarithmic plots of the *I*-*V* characteristics for both of the structures. These curves are characterized by three distinct linear regions each indicating different conducting mechanisms. The curves present voltage dependence followed by power law dependence at higher voltage regions. This behavior can be attributed to space-charge-limited current (SCLC) [23, 24]. From the figure, it can be seen that the graphs have three distinct regions that change in the form of *I* *α* *V* ^*m*^. It is obvious that the exponent *m* values are calculated from the slopes of these three different regions. The first region is characterized as the ohmic region (low bias region). The *m* values are 2.56 and 2.37 for the structures grown by CBD and sol-gel methods, respectively. Although these values are bigger than the unity, they are still close to the ohmic region, but in the second region, the *m* values were increased to 4.30 and 4.81. The high *m* value indicates that the SCLC mechanism is controlled by the presence of traps within the band gap of the CuO films. The third regions (high bias region) also indicate the SCLC mechanism, but in this case the *m* values decrease to lower values (2.60 for CBD and 3.26 for sol-gel methods). This is because the devices approach the “trap-filled” limits [23]. 

## 4. Conclusion 

In the present work, we have fabricated Au/CuO/p-Si/Al MIS structures by using two different methods. The morphological and structural properties of the CuO films were investigated through SEM and XRD methods. The electronic parameters such as ideality factor, barrier height, and series resistance of the Schottky diodes were calculated and compared from the forward bias *I*-*V* and Norde's plots. The SCLC theory was successfully applied to the produced diodes.

- From the SEM investigations, it was observed that the film grown by CBD method was found to be continuous and has distributed large grains covering the entire surface while the sol-gel grown structures were not continuous. According to the morphological investigations, we attributed that this discreetness affected the ideality factors and the barrier heights of our diodes.
- The XRD diffraction patterns of both sample matched very well with the reference PDF cards. It was deduced that the crystallization was stronger in CBD method than in sol-gel method.
- From the electrical characterizations, it was found that the ideality factors were deviated from the unity which can be related to current mechanism of the structure, barrier height inhomogeneity, series resistance, and image force levering which is voltage dependent and/or an interface layer. Also it was determined that both the series resistance and barrier height values of the structure grown by CBD method are lower than the structure grown by sol-gel method. From these results, it was shown that the deposition method and the morphology of CuO layer have significant effects on the performance of the devices.

This paper highlights an investigation on the Schottky diode using CuO as the interface layer. The performance and quality of the Schottky diodes are especially dependent on the fabrication conditions and formation of interlayer which strongly affect on device parameters such as ideality factor, barrier height and serial resistance. Some of these device parameters can be manipulated by modified interfacial layers that are grown in between the metal and semiconductor. We concluded that producing CuO interlayer via chemical bath deposition method is a convenient and effective way to modify the device parameters of the diodes. 

## Figures and Tables

**Table 1:** Structural parameters of the CuO films.

Deposition method	Grain size	Microstrain	Dislocation density
	(*D*) (nm)	(*ε*) × 10^−4^	(*ρ*) (cm^2^)
Sol-gel	13.83	21.5	4.67 × 10^15^
CBD	15.02	36.1	2.83 × 10^16^

**Table 2:** Ideality factor, barrier height, and series resistance values of the MIS structures.

	*I*-*V*		Norde's functions	
	*n*	Φ_*B*_ (eV)	Φ_*B*_ (eV)	*R*_*S*_ (kΩ)
Sol-gel	1.58	0.79	0.80	410
CBD	2.80	0.79	0.76	119
